# Bruceine A

**DOI:** 10.1107/S1600536810007646

**Published:** 2010-03-17

**Authors:** Xue-Huan Feng, Yan-Ning Zhang, Wei-Zhi He, Lan Zhang, Hong-Yun Jiang

**Affiliations:** a State Key Laboratory for Biology of Plant Diseases and Insect Pests, Institute of Plant Protection, Chinese Academy of Agricultural Sciences, Beijing 100193, People’s Republic of China

## Abstract

The title compound, C_26_H_34_O_11_, known as bruceine A, is a natural quassinoid extracted from the dried fruits of *Brucea javanica*. Its structure consists of five fused rings including an oxygen-containing heterocyclic ring and a lactone ring. Two intra­molecular O—H⋯O links help to establish the mol­ecular conformation. In the crystal, O—H⋯O hydrogen bonds connect the mol­ecules.

## Related literature

For medicinal and pharmacological background to *Brucea javanica* and its extracts, see: Anderson *et al.* (1991 ▶); Bawm *et al.* (2008 ▶); Elkhateeb *et al.* (2008 ▶); Klocke *et al.* (1985 ▶); Leskinen *et al.* (1984 ▶); Nakao *et al.* (2009 ▶); O’Neill *et al.* (1987 ▶); Odjo *et al.* (1981 ▶); Pan *et al.* (2009 ▶); Pavanand *et al.* (1986 ▶); Subeki *et al.* (2007 ▶).
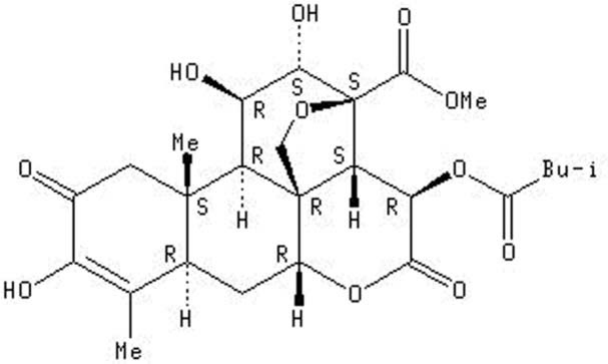


## Experimental

### 

#### Crystal data

C_26_H_34_O_11_
*M* *_r_* = 522.53
Orthorhombic, 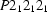

*a* = 9.0337 (12) Å
*b* = 10.167 (3) Å
*c* = 26.9122 (11) Å
*V* = 2471.8 (8) Å^3^
*Z* = 4
Cu *K*α radiation
μ = 0.92 mm^−1^
*T* = 173 K
0.44 × 0.30 × 0.14 mm

#### Data collection

Rigaku R-AXIS RAPID IP area-detector diffractometer
Absorption correction: multi-scan (*ABSCOR*; Higashi, 1995 ▶) *T*_min_ = 0.687, *T*_max_ = 0.882
17108 measured reflections
4478 independent reflections
4051 reflections with *I* > 2σ(*I*)
*R*_int_ = 0.044

#### Refinement

*R*[*F* ^2^ > 2σ(*F* ^2^)] = 0.044
*wR*(*F* ^2^) = 0.098
*S* = 1.13
4478 reflections
340 parameters
H-atom parameters constrained
Δρ_max_ = 0.21 e Å^−3^
Δρ_min_ = −0.21 e Å^−3^
Absolute structure: Flack (1983 ▶), 1887 Friedel pairs
Flack parameter: −0.3 (2)

### 

Data collection: *RAPID-AUTO* (Rigaku, 2001 ▶); cell refinement: *RAPID-AUTO*; data reduction: *RAPID-AUTO*; program(s) used to solve structure: *SHELXS97* (Sheldrick, 2008 ▶); program(s) used to refine structure: *SHELXL97* (Sheldrick, 2008 ▶); molecular graphics: *XP* in *SHELXTL* (Sheldrick, 2008 ▶); software used to prepare material for publication: *SHELXL97*.

## Supplementary Material

Crystal structure: contains datablocks I, global. DOI: 10.1107/S1600536810007646/hb5331sup1.cif

Structure factors: contains datablocks I. DOI: 10.1107/S1600536810007646/hb5331Isup2.hkl

Additional supplementary materials:  crystallographic information; 3D view; checkCIF report

## Figures and Tables

**Table 1:** Hydrogen-bond geometry (Å, °)

*D*—H⋯*A*	*D*—H	H⋯*A*	*D*⋯*A*	*D*—H⋯*A*
O2—H2*A*⋯O9^i^	0.84	2.11	2.870 (3)	150
O2—H2*A*⋯O1	0.84	2.28	2.718 (3)	113
O5—H5*A*⋯O10^ii^	0.84	2.17	2.874 (2)	142
O5—H5*A*⋯O11	0.84	2.19	2.797 (2)	129
O6—H6*A*⋯O4^iii^	0.84	1.97	2.799 (3)	171

Symmetry codes: (i) 
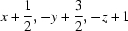
; (ii) 
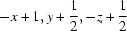
; (iii) 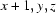.

**Fig. 1.**
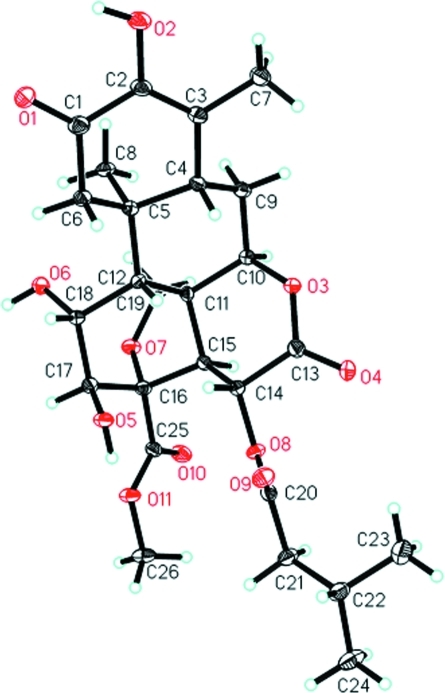
Ellipsoid plot.

## supplementary crystallographic information

### Comment

The bitter fruits of medicinal plant *Brucea javanica *(L.) Merr. Simaroubaceae is widely used in traditional medicine for various ailments (Bawm *et al.*, 2008). Bruceine A, a natural quassinoid compound extracted from the fruits of *B. javanica* (L.) Merr., with a wide spectrum of biological effects, such as having potential antibabesial,antitrypanosomal, antimalarial and cytotoxicity against human cancer cell lines (Pan *et al.*, 2009; Nakao *et al.*, 2009; Elkhateeb *et al.*, 2008; Subeki *et al.*, 2007; Anderson *et al.*,1991; O'Neill *et al.*,1987; Pavanand *et al.*,1986). Bruceine A was also shown to have insecticidal, antifeedant, and growth inhibitory effects against the tobacco budworm, *Heliothis virescens *(F.), and the fall armyworm, *Spodoptera frugiperda* (Klocke *et al.*, 1985), and strong antifeedant activity on the 3rd larvae of Locusta migratoria migratorioides R and F (Orthoptera, Acrididae) (Odjo *et al.*,1981) and the 4th instar larvae of Mexican bean beetle (Epilachna varivestis Mulsant) (Leskinen *et al.*,1984).

As part of our studies in this area, we have isolated the title compound (I), which has potential insecticidal activity against *Spodoptera exigua* (Lepidoptera: Noctuidae). The crystal structure of the title compound is shown in Fig. 1.

### Experimental

The dried fruits of *B. javanica* were extracted with 80% ethanol for three days. Then the solution was filtered and removed into vacuo, and extracted with CHCl_3_ to give aqueous and CHCl_3_ layers. The CHCl_3_ layer was chromatographed on a silica gel column, and eluted successively with different MeOH–CHCl_3_ ratios. The MeOH–CHCl_3_ (20:80) eluate was evaporated to yield a residue, which was subjected to column chromatography on silica gel, eluted with hexane–EtOAc (50:50), to give the title compound (Bruceine A). The title compound was dissolved in acetone (20 ml) at room temperature, Colourless plates of (I) were obtained through slow evaporation after two weeks.

### Refinement

All the hydrogen atoms were placed at their geometrical positions (C—H = 0.93–0.98Å; O–H = 0.84Å) and refined as riding with *U*_iso_(H) = 1.2–1.5*U*_eq_(C,O).

### Crystal data

**Table:**

C_26_H_34_O_11_	*F*(000) = 1112
*M**_r_* = 522.53	*D*_x_ = 1.404 Mg m^−^^3^
Orthorhombic, *P*2_1_2_1_2_1_	Cu *K*α radiation, λ = 1.54186 Å
Hall symbol: P 2ac 2ab	Cell parameters from 657 reflections
*a* = 9.0337 (12) Å	θ = 3.1–66.2°
*b* = 10.167 (3) Å	µ = 0.92 mm^−^^1^
*c* = 26.9122 (11) Å	*T* = 173 K
*V* = 2471.8 (8) Å^3^	Plate, colourless
*Z* = 4	0.44 × 0.30 × 0.14 mm

### Data collection

**Table:**

Rigaku R-AXIS RAPID IP area-detector diffractometer	4478 independent reflections
Radiation source: rotating anode	4051 reflections with *I* > 2σ(*I*)
graphite	*R*_int_ = 0.044
ω scans at fixed χ = 45°	θ_max_ = 68.2°, θ_min_ = 3.3°
Absorption correction: multi-scan (*ABSCOR*; Higashi, 1995)	*h* = −10→10
*T*_min_ = 0.687, *T*_max_ = 0.882	*k* = −11→12
17108 measured reflections	*l* = −32→32

### Refinement

**Table:**

Refinement on *F*^2^	Hydrogen site location: inferred from neighbouring sites
Least-squares matrix: full	H-atom parameters constrained
*R*[*F*^2^ > 2σ(*F*^2^)] = 0.044	*w* = 1/[σ^2^(*F*_o_^2^) + (0.0199*P*)^2^ + 1.6367*P*] where *P* = (*F*_o_^2^ + 2*F*_c_^2^)/3
*wR*(*F*^2^) = 0.098	(Δ/σ)_max_ < 0.001
*S* = 1.13	Δρ_max_ = 0.21 e Å^−^^3^
4478 reflections	Δρ_min_ = −0.21 e Å^−^^3^
340 parameters	Extinction correction: *SHELXL97* (Sheldrick, 2008), Fc^*^=kFc[1+0.001xFc^2^λ^3^/sin(2θ)]^-1/4^
0 restraints	Extinction coefficient: 0.00127 (11)
Primary atom site location: structure-invariant direct methods	Absolute structure: Flack (1983), 1887 Friedel pairs
Secondary atom site location: difference Fourier map	Flack parameter: −0.3 (2)

### Special details

**Table:**

Geometry. All esds (except the esd in the dihedral angle between two l.s. planes) are estimated using the full covariance matrix. The cell esds are taken into account individually in the estimation of esds in distances, angles and torsion angles; correlations between esds in cell parameters are only used when they are defined by crystal symmetry. An approximate (isotropic) treatment of cell esds is used for estimating esds involving l.s. planes.
Refinement. Refinement of *F*^2^ against ALL reflections. The weighted *R*-factor wR and goodness of fit *S* are based on *F*^2^, conventional *R*-factors *R* are based on *F*, with *F* set to zero for negative *F*^2^. The threshold expression of *F*^2^ > σ(*F*^2^) is used only for calculating *R*-factors(gt) etc. and is not relevant to the choice of reflections for refinement. *R*-factors based on *F*^2^ are statistically about twice as large as those based on *F*, and *R*- factors based on ALL data will be even larger.

### Fractional atomic coordinates and isotropic or equivalent isotropic displacement parameters (Å^2^)

**Table:**

	*x*	*y*	*z*	*U*_iso_*/*U*_eq_	
O1	0.7087 (2)	0.7346 (2)	0.58066 (7)	0.0362 (5)	
O2	0.4645 (2)	0.6705 (2)	0.63450 (7)	0.0372 (5)	
H2A	0.5479	0.6962	0.6444	0.056*	
O3	0.1686 (2)	0.4600 (2)	0.42513 (6)	0.0261 (5)	
O4	0.0274 (2)	0.5360 (2)	0.36490 (7)	0.0308 (5)	
O5	0.5773 (2)	0.64791 (18)	0.32956 (6)	0.0241 (4)	
H5A	0.5610	0.6436	0.2989	0.036*	
O6	0.7799 (2)	0.4626 (2)	0.42098 (7)	0.0292 (5)	
H6A	0.8595	0.4779	0.4060	0.044*	
O7	0.6075 (2)	0.29438 (18)	0.35876 (6)	0.0248 (5)	
O8	0.2473 (2)	0.51955 (19)	0.29566 (6)	0.0224 (4)	
O9	0.1862 (2)	0.73521 (19)	0.29790 (7)	0.0284 (5)	
O10	0.4926 (2)	0.26506 (19)	0.26475 (6)	0.0308 (5)	
O11	0.5696 (2)	0.47209 (19)	0.24965 (6)	0.0274 (5)	
C1	0.6047 (4)	0.6833 (3)	0.55833 (10)	0.0283 (7)	
C2	0.4716 (3)	0.6417 (3)	0.58506 (10)	0.0268 (6)	
C3	0.3604 (3)	0.5767 (3)	0.56249 (10)	0.0238 (6)	
C4	0.3724 (3)	0.5357 (3)	0.50844 (9)	0.0209 (6)	
H4A	0.3181	0.6035	0.4886	0.025*	
C5	0.5338 (3)	0.5333 (3)	0.48864 (9)	0.0200 (5)	
C6	0.6068 (3)	0.6646 (3)	0.50295 (9)	0.0237 (6)	
H6B	0.7104	0.6658	0.4909	0.028*	
H6C	0.5532	0.7380	0.4868	0.028*	
C7	0.2220 (3)	0.5461 (3)	0.59057 (10)	0.0306 (7)	
H7A	0.2139	0.6051	0.6192	0.046*	
H7B	0.2251	0.4547	0.6021	0.046*	
H7C	0.1362	0.5585	0.5688	0.046*	
C8	0.6234 (3)	0.4226 (3)	0.51357 (10)	0.0245 (7)	
H8A	0.6182	0.4324	0.5498	0.037*	
H8B	0.7269	0.4278	0.5028	0.037*	
H8C	0.5821	0.3371	0.5040	0.037*	
C9	0.2980 (3)	0.4027 (3)	0.49735 (9)	0.0256 (6)	
H9A	0.3539	0.3314	0.5139	0.031*	
H9B	0.1961	0.4029	0.5108	0.031*	
C10	0.2930 (3)	0.3772 (3)	0.44191 (9)	0.0202 (6)	
H10A	0.2640	0.2832	0.4369	0.024*	
C11	0.4358 (3)	0.4013 (3)	0.41255 (9)	0.0180 (6)	
C12	0.5213 (3)	0.5248 (3)	0.43077 (8)	0.0184 (5)	
H12A	0.4572	0.6009	0.4212	0.022*	
C13	0.1512 (3)	0.5072 (3)	0.37875 (10)	0.0241 (6)	
C14	0.2882 (3)	0.5266 (3)	0.34730 (9)	0.0204 (6)	
H14A	0.3358	0.6131	0.3549	0.024*	
C15	0.3934 (3)	0.4154 (3)	0.35733 (9)	0.0187 (6)	
H15A	0.3408	0.3326	0.3478	0.022*	
C16	0.5477 (3)	0.4080 (3)	0.33405 (9)	0.0208 (6)	
C17	0.6442 (3)	0.5287 (3)	0.34586 (9)	0.0221 (6)	
H17A	0.7428	0.5185	0.3294	0.026*	
C18	0.6665 (3)	0.5457 (3)	0.40194 (9)	0.0215 (6)	
H18A	0.6984	0.6387	0.4077	0.026*	
C19	0.5370 (3)	0.2805 (3)	0.40671 (9)	0.0224 (6)	
H19A	0.6121	0.2782	0.4335	0.027*	
H19B	0.4783	0.1983	0.4080	0.027*	
C20	0.1948 (3)	0.6338 (3)	0.27507 (10)	0.0228 (6)	
C21	0.1545 (3)	0.6143 (3)	0.22154 (10)	0.0286 (7)	
H21A	0.1314	0.5203	0.2158	0.034*	
H21B	0.2403	0.6377	0.2004	0.034*	
C22	0.0221 (3)	0.6972 (3)	0.20635 (10)	0.0318 (7)	
H22A	0.0442	0.7913	0.2143	0.038*	
C23	−0.1148 (4)	0.6563 (5)	0.23499 (13)	0.0614 (12)	
H23A	−0.0980	0.6696	0.2706	0.092*	
H23B	−0.1356	0.5632	0.2286	0.092*	
H23C	−0.1992	0.7097	0.2242	0.092*	
C24	−0.0041 (4)	0.6853 (3)	0.15038 (10)	0.0406 (8)	
H24A	0.0846	0.7142	0.1325	0.061*	
H24B	−0.0882	0.7407	0.1408	0.061*	
H24C	−0.0255	0.5935	0.1420	0.061*	
C25	0.5346 (3)	0.3717 (3)	0.27880 (9)	0.0219 (6)	
C26	0.5432 (4)	0.4542 (3)	0.19654 (9)	0.0334 (7)	
H26A	0.5425	0.5402	0.1800	0.050*	
H26B	0.4474	0.4109	0.1916	0.050*	
H26C	0.6220	0.3996	0.1823	0.050*	

### Atomic displacement parameters (Å^2^)

**Table:**

	*U*^11^	*U*^22^	*U*^33^	*U*^12^	*U*^13^	*U*^23^
O1	0.0367 (13)	0.0451 (13)	0.0268 (11)	−0.0157 (11)	−0.0012 (10)	−0.0098 (10)
O2	0.0447 (13)	0.0469 (13)	0.0201 (10)	−0.0177 (12)	0.0047 (10)	−0.0092 (10)
O3	0.0206 (10)	0.0399 (12)	0.0177 (10)	0.0006 (9)	0.0003 (8)	0.0049 (9)
O4	0.0181 (10)	0.0443 (12)	0.0299 (11)	0.0001 (10)	−0.0005 (9)	0.0046 (10)
O5	0.0320 (11)	0.0245 (10)	0.0160 (9)	−0.0028 (9)	−0.0003 (8)	0.0018 (8)
O6	0.0193 (10)	0.0444 (13)	0.0239 (10)	0.0039 (10)	0.0007 (8)	0.0059 (10)
O7	0.0284 (11)	0.0261 (11)	0.0198 (10)	0.0060 (9)	0.0016 (8)	0.0028 (8)
O8	0.0254 (10)	0.0268 (10)	0.0150 (9)	0.0032 (9)	−0.0046 (8)	−0.0005 (8)
O9	0.0328 (12)	0.0266 (11)	0.0258 (10)	0.0023 (10)	−0.0041 (9)	−0.0026 (9)
O10	0.0412 (13)	0.0299 (11)	0.0214 (10)	−0.0062 (10)	0.0040 (9)	−0.0042 (8)
O11	0.0379 (12)	0.0291 (10)	0.0152 (9)	−0.0013 (10)	−0.0013 (8)	0.0012 (8)
C1	0.0361 (18)	0.0262 (16)	0.0224 (15)	−0.0050 (14)	0.0001 (13)	−0.0037 (12)
C2	0.0341 (16)	0.0287 (15)	0.0177 (13)	−0.0039 (14)	0.0043 (13)	−0.0059 (12)
C3	0.0287 (16)	0.0230 (14)	0.0197 (14)	−0.0008 (12)	0.0042 (12)	−0.0010 (11)
C4	0.0224 (14)	0.0231 (14)	0.0171 (13)	−0.0025 (13)	0.0026 (11)	0.0013 (12)
C5	0.0216 (13)	0.0216 (13)	0.0168 (12)	−0.0007 (13)	0.0000 (11)	0.0028 (11)
C6	0.0269 (16)	0.0263 (15)	0.0179 (13)	−0.0046 (13)	0.0005 (12)	−0.0038 (12)
C7	0.0326 (16)	0.0345 (17)	0.0247 (15)	−0.0085 (15)	0.0045 (13)	−0.0031 (13)
C8	0.0280 (16)	0.0300 (16)	0.0155 (13)	0.0053 (13)	−0.0012 (12)	−0.0007 (12)
C9	0.0289 (15)	0.0300 (16)	0.0178 (14)	−0.0055 (14)	0.0016 (12)	0.0006 (12)
C10	0.0196 (14)	0.0233 (15)	0.0176 (13)	−0.0001 (12)	0.0003 (11)	0.0008 (11)
C11	0.0153 (13)	0.0200 (13)	0.0187 (13)	−0.0029 (11)	0.0003 (11)	−0.0014 (11)
C12	0.0178 (13)	0.0207 (13)	0.0166 (12)	−0.0013 (12)	−0.0024 (11)	0.0011 (11)
C13	0.0219 (14)	0.0268 (15)	0.0237 (14)	−0.0006 (13)	0.0022 (11)	−0.0010 (12)
C14	0.0179 (13)	0.0270 (15)	0.0163 (13)	0.0000 (13)	−0.0009 (10)	0.0006 (12)
C15	0.0182 (13)	0.0208 (14)	0.0169 (13)	−0.0026 (12)	0.0016 (11)	−0.0009 (11)
C16	0.0219 (14)	0.0231 (14)	0.0173 (13)	0.0035 (12)	−0.0002 (11)	0.0031 (11)
C17	0.0225 (14)	0.0243 (14)	0.0193 (14)	−0.0033 (13)	0.0000 (11)	−0.0005 (12)
C18	0.0210 (14)	0.0285 (15)	0.0151 (13)	−0.0016 (13)	−0.0008 (11)	0.0019 (12)
C19	0.0266 (15)	0.0239 (14)	0.0167 (13)	0.0018 (13)	−0.0010 (12)	0.0033 (11)
C20	0.0179 (14)	0.0260 (15)	0.0243 (14)	0.0021 (12)	0.0027 (12)	0.0045 (12)
C21	0.0298 (16)	0.0356 (17)	0.0204 (14)	0.0058 (15)	−0.0047 (12)	−0.0011 (13)
C22	0.0305 (16)	0.0397 (17)	0.0252 (15)	0.0077 (15)	−0.0054 (13)	0.0029 (13)
C23	0.030 (2)	0.111 (4)	0.043 (2)	0.005 (2)	−0.0012 (16)	0.010 (2)
C24	0.046 (2)	0.046 (2)	0.0298 (16)	0.0170 (17)	−0.0123 (15)	0.0003 (15)
C25	0.0223 (14)	0.0244 (14)	0.0191 (13)	0.0011 (13)	0.0032 (12)	0.0013 (11)
C26	0.0490 (19)	0.0343 (17)	0.0169 (14)	0.0012 (17)	−0.0033 (14)	0.0016 (12)

### Geometric parameters (Å, °)

**Table:**

O1—C1	1.231 (3)	C9—H9A	0.9900
O2—C2	1.364 (3)	C9—H9B	0.9900
O2—H2A	0.8401	C10—C11	1.532 (3)
O3—C13	1.346 (3)	C10—H10A	1.0000
O3—C10	1.475 (3)	C11—C19	1.539 (4)
O4—C13	1.215 (3)	C11—C15	1.541 (3)
O5—C17	1.424 (3)	C11—C12	1.554 (3)
O5—H5A	0.8401	C12—C18	1.539 (3)
O6—C18	1.423 (3)	C12—H12A	1.0000
O6—H6A	0.8400	C13—C14	1.512 (4)
O7—C16	1.438 (3)	C14—C15	1.502 (4)
O7—C19	1.446 (3)	C14—H14A	1.0000
O8—C20	1.371 (3)	C15—C16	1.530 (4)
O8—C14	1.440 (3)	C15—H15A	1.0000
O9—C20	1.203 (3)	C16—C25	1.536 (3)
O10—C25	1.210 (3)	C16—C17	1.539 (4)
O11—C25	1.325 (3)	C17—C18	1.532 (3)
O11—C26	1.460 (3)	C17—H17A	1.0000
C1—C2	1.463 (4)	C18—H18A	1.0000
C1—C6	1.503 (3)	C19—H19A	0.9900
C2—C3	1.347 (4)	C19—H19B	0.9900
C3—C7	1.494 (4)	C20—C21	1.499 (4)
C3—C4	1.517 (3)	C21—C22	1.519 (4)
C4—C9	1.539 (4)	C21—H21A	0.9900
C4—C5	1.553 (4)	C21—H21B	0.9900
C4—H4A	1.0000	C22—C23	1.515 (4)
C5—C6	1.539 (4)	C22—C24	1.529 (4)
C5—C8	1.540 (4)	C22—H22A	1.0000
C5—C12	1.564 (3)	C23—H23A	0.9800
C6—H6B	0.9900	C23—H23B	0.9800
C6—H6C	0.9900	C23—H23C	0.9800
C7—H7A	0.9800	C24—H24A	0.9800
C7—H7B	0.9800	C24—H24B	0.9800
C7—H7C	0.9800	C24—H24C	0.9800
C8—H8A	0.9800	C26—H26A	0.9800
C8—H8B	0.9800	C26—H26B	0.9800
C8—H8C	0.9800	C26—H26C	0.9800
C9—C10	1.515 (3)		
			
C2—O2—H2A	109.5	O8—C14—C15	107.4 (2)
C13—O3—C10	125.2 (2)	O8—C14—C13	108.9 (2)
C17—O5—H5A	109.5	C15—C14—C13	108.6 (2)
C18—O6—H6A	109.5	O8—C14—H14A	110.6
C16—O7—C19	109.04 (19)	C15—C14—H14A	110.6
C20—O8—C14	115.9 (2)	C13—C14—H14A	110.6
C25—O11—C26	116.4 (2)	C14—C15—C16	122.7 (2)
O1—C1—C2	120.6 (2)	C14—C15—C11	113.6 (2)
O1—C1—C6	121.8 (3)	C16—C15—C11	99.44 (19)
C2—C1—C6	117.5 (2)	C14—C15—H15A	106.7
C3—C2—O2	120.7 (3)	C16—C15—H15A	106.7
C3—C2—C1	122.2 (2)	C11—C15—H15A	106.7
O2—C2—C1	117.1 (2)	O7—C16—C15	101.1 (2)
C2—C3—C7	119.9 (2)	O7—C16—C25	106.5 (2)
C2—C3—C4	120.9 (2)	C15—C16—C25	109.8 (2)
C7—C3—C4	119.2 (2)	O7—C16—C17	109.4 (2)
C3—C4—C9	113.3 (2)	C15—C16—C17	113.1 (2)
C3—C4—C5	113.6 (2)	C25—C16—C17	115.8 (2)
C9—C4—C5	109.2 (2)	O5—C17—C18	105.3 (2)
C3—C4—H4A	106.7	O5—C17—C16	112.0 (2)
C9—C4—H4A	106.7	C18—C17—C16	111.6 (2)
C5—C4—H4A	106.7	O5—C17—H17A	109.3
C6—C5—C8	107.5 (2)	C18—C17—H17A	109.3
C6—C5—C4	107.6 (2)	C16—C17—H17A	109.3
C8—C5—C4	110.8 (2)	O6—C18—C17	112.5 (2)
C6—C5—C12	109.2 (2)	O6—C18—C12	110.5 (2)
C8—C5—C12	115.6 (2)	C17—C18—C12	111.6 (2)
C4—C5—C12	105.9 (2)	O6—C18—H18A	107.3
C1—C6—C5	110.6 (2)	C17—C18—H18A	107.3
C1—C6—H6B	109.5	C12—C18—H18A	107.3
C5—C6—H6B	109.5	O7—C19—C11	105.9 (2)
C1—C6—H6C	109.5	O7—C19—H19A	110.5
C5—C6—H6C	109.5	C11—C19—H19A	110.5
H6B—C6—H6C	108.1	O7—C19—H19B	110.5
C3—C7—H7A	109.5	C11—C19—H19B	110.5
C3—C7—H7B	109.5	H19A—C19—H19B	108.7
H7A—C7—H7B	109.5	O9—C20—O8	122.8 (2)
C3—C7—H7C	109.5	O9—C20—C21	126.1 (3)
H7A—C7—H7C	109.5	O8—C20—C21	111.1 (2)
H7B—C7—H7C	109.5	C20—C21—C22	112.1 (2)
C5—C8—H8A	109.5	C20—C21—H21A	109.2
C5—C8—H8B	109.5	C22—C21—H21A	109.2
H8A—C8—H8B	109.5	C20—C21—H21B	109.2
C5—C8—H8C	109.5	C22—C21—H21B	109.2
H8A—C8—H8C	109.5	H21A—C21—H21B	107.9
H8B—C8—H8C	109.5	C23—C22—C21	110.7 (3)
C10—C9—C4	110.8 (2)	C23—C22—C24	110.7 (3)
C10—C9—H9A	109.5	C21—C22—C24	110.1 (2)
C4—C9—H9A	109.5	C23—C22—H22A	108.5
C10—C9—H9B	109.5	C21—C22—H22A	108.5
C4—C9—H9B	109.5	C24—C22—H22A	108.5
H9A—C9—H9B	108.1	C22—C23—H23A	109.5
O3—C10—C9	103.1 (2)	C22—C23—H23B	109.5
O3—C10—C11	113.1 (2)	H23A—C23—H23B	109.5
C9—C10—C11	117.1 (2)	C22—C23—H23C	109.5
O3—C10—H10A	107.7	H23A—C23—H23C	109.5
C9—C10—H10A	107.7	H23B—C23—H23C	109.5
C11—C10—H10A	107.7	C22—C24—H24A	109.5
C10—C11—C19	115.2 (2)	C22—C24—H24B	109.5
C10—C11—C15	107.7 (2)	H24A—C24—H24B	109.5
C19—C11—C15	97.09 (19)	C22—C24—H24C	109.5
C10—C11—C12	112.6 (2)	H24A—C24—H24C	109.5
C19—C11—C12	112.4 (2)	H24B—C24—H24C	109.5
C15—C11—C12	110.6 (2)	O10—C25—O11	125.5 (2)
C18—C12—C11	112.1 (2)	O10—C25—C16	122.8 (2)
C18—C12—C5	115.6 (2)	O11—C25—C16	111.7 (2)
C11—C12—C5	113.3 (2)	O11—C26—H26A	109.5
C18—C12—H12A	104.8	O11—C26—H26B	109.5
C11—C12—H12A	104.8	H26A—C26—H26B	109.5
C5—C12—H12A	104.8	O11—C26—H26C	109.5
O4—C13—O3	118.6 (2)	H26A—C26—H26C	109.5
O4—C13—C14	123.4 (2)	H26B—C26—H26C	109.5
O3—C13—C14	118.0 (2)		
			
O1—C1—C2—C3	−175.1 (3)	O4—C13—C14—C15	−144.5 (3)
C6—C1—C2—C3	6.7 (4)	O3—C13—C14—C15	38.1 (3)
O1—C1—C2—O2	4.4 (4)	O8—C14—C15—C16	66.1 (3)
C6—C1—C2—O2	−173.8 (2)	C13—C14—C15—C16	−176.3 (2)
O2—C2—C3—C7	5.4 (4)	O8—C14—C15—C11	−174.3 (2)
C1—C2—C3—C7	−175.1 (3)	C13—C14—C15—C11	−56.7 (3)
O2—C2—C3—C4	−175.6 (3)	C10—C11—C15—C14	59.6 (3)
C1—C2—C3—C4	3.9 (4)	C19—C11—C15—C14	178.9 (2)
C2—C3—C4—C9	144.4 (3)	C12—C11—C15—C14	−63.8 (3)
C7—C3—C4—C9	−36.6 (4)	C10—C11—C15—C16	−168.3 (2)
C2—C3—C4—C5	18.9 (4)	C19—C11—C15—C16	−49.0 (2)
C7—C3—C4—C5	−162.1 (2)	C12—C11—C15—C16	68.3 (3)
C3—C4—C5—C6	−49.3 (3)	C19—O7—C16—C15	−27.3 (2)
C9—C4—C5—C6	−176.9 (2)	C19—O7—C16—C25	−141.9 (2)
C3—C4—C5—C8	67.9 (3)	C19—O7—C16—C17	92.2 (2)
C9—C4—C5—C8	−59.7 (3)	C14—C15—C16—O7	174.3 (2)
C3—C4—C5—C12	−166.0 (2)	C11—C15—C16—O7	48.2 (2)
C9—C4—C5—C12	66.4 (3)	C14—C15—C16—C25	−73.5 (3)
O1—C1—C6—C5	142.6 (3)	C11—C15—C16—C25	160.4 (2)
C2—C1—C6—C5	−39.3 (4)	C14—C15—C16—C17	57.4 (3)
C8—C5—C6—C1	−60.5 (3)	C11—C15—C16—C17	−68.7 (3)
C4—C5—C6—C1	58.9 (3)	O7—C16—C17—O5	−170.0 (2)
C12—C5—C6—C1	173.4 (2)	C15—C16—C17—O5	−58.2 (3)
C3—C4—C9—C10	171.4 (2)	C25—C16—C17—O5	69.7 (3)
C5—C4—C9—C10	−60.9 (3)	O7—C16—C17—C18	−52.3 (3)
C13—O3—C10—C9	155.6 (2)	C15—C16—C17—C18	59.5 (3)
C13—O3—C10—C11	28.2 (3)	C25—C16—C17—C18	−172.6 (2)
C4—C9—C10—O3	−78.0 (3)	O5—C17—C18—O6	−157.1 (2)
C4—C9—C10—C11	46.9 (3)	C16—C17—C18—O6	81.1 (3)
O3—C10—C11—C19	−148.5 (2)	O5—C17—C18—C12	78.1 (3)
C9—C10—C11—C19	91.8 (3)	C16—C17—C18—C12	−43.7 (3)
O3—C10—C11—C15	−41.5 (3)	C11—C12—C18—O6	−80.7 (3)
C9—C10—C11—C15	−161.1 (2)	C5—C12—C18—O6	51.2 (3)
O3—C10—C11—C12	80.7 (3)	C11—C12—C18—C17	45.2 (3)
C9—C10—C11—C12	−38.9 (3)	C5—C12—C18—C17	177.1 (2)
C10—C11—C12—C18	178.5 (2)	C16—O7—C19—C11	−4.5 (3)
C19—C11—C12—C18	46.3 (3)	C10—C11—C19—O7	147.0 (2)
C15—C11—C12—C18	−61.0 (3)	C15—C11—C19—O7	33.7 (2)
C10—C11—C12—C5	45.4 (3)	C12—C11—C19—O7	−82.2 (2)
C19—C11—C12—C5	−86.8 (3)	C14—O8—C20—O9	1.7 (4)
C15—C11—C12—C5	165.9 (2)	C14—O8—C20—C21	−179.4 (2)
C6—C5—C12—C18	53.9 (3)	O9—C20—C21—C22	−35.3 (4)
C8—C5—C12—C18	−67.3 (3)	O8—C20—C21—C22	145.8 (2)
C4—C5—C12—C18	169.6 (2)	C20—C21—C22—C23	−63.5 (4)
C6—C5—C12—C11	−174.7 (2)	C20—C21—C22—C24	173.9 (3)
C8—C5—C12—C11	64.1 (3)	C26—O11—C25—O10	5.2 (4)
C4—C5—C12—C11	−59.1 (3)	C26—O11—C25—C16	−172.5 (2)
C10—O3—C13—O4	156.4 (3)	O7—C16—C25—O10	40.6 (4)
C10—O3—C13—C14	−26.1 (4)	C15—C16—C25—O10	−68.0 (3)
C20—O8—C14—C15	−158.5 (2)	C17—C16—C25—O10	162.5 (3)
C20—O8—C14—C13	84.2 (3)	O7—C16—C25—O11	−141.6 (2)
O4—C13—C14—O8	−27.9 (4)	C15—C16—C25—O11	109.8 (3)
O3—C13—C14—O8	154.7 (2)	C17—C16—C25—O11	−19.7 (3)

### Hydrogen-bond geometry (Å, °)

**Table:**

*D*—H···*A*	*D*—H	H···*A*	*D*···*A*	*D*—H···*A*
O2—H2A···O9^i^	0.84	2.11	2.870 (3)	150
O2—H2A···O1	0.84	2.28	2.718 (3)	113
O5—H5A···O10^ii^	0.84	2.17	2.874 (2)	142
O5—H5A···O11	0.84	2.19	2.797 (2)	129
O6—H6A···O4^iii^	0.84	1.97	2.799 (3)	171

Symmetry codes: (i) *x*+1/2, −*y*+3/2, −*z*+1; (ii) −*x*+1, *y*+1/2, −*z*+1/2; (iii) *x*+1, *y*, *z*.
